# Comment on “Fast and accurate electromagnetic field calculation for substrate-supported metasurfaces using the discrete dipole approximation”

**DOI:** 10.1515/nanoph-2023-0849

**Published:** 2024-05-08

**Authors:** Patrick C. Chaumet

**Affiliations:** Aix Marseille Univ, CNRS, Centrale Med, Institut Fresnel, Marseille, France

**Keywords:** DDA, Green tensor, multilayered

## Abstract

The article entitled “Fast and accurate electromagnetic field calculation for substrate-supported metasurfaces using the discrete dipole approximation (DDA)” written by W. Liu and E. McLeod presents a method for computing the Green function in presence of a substrate or multilayer in an efficient way. Unfortunately, the proposed method has been known for more than twenty years and significantly ameliorated. It can be found in its optimized version in some open-source DDA codes. In this comment, we recall the different approaches (and related papers) addressing the computation of the Green function of a multilayered system. We show that they address all the points raised by Liu and McLeod in their paper and provide more efficient solutions to their computational issues.

In a recent paper Liu and McLeod [1] present a fast and accurate calculation of the Green tensor for a substrate or multilayered system. However, their article overlooks the work that has been done on this subject in the last few decades.

The first article to provide the expression of the Green’s tensor for a gap (and therefore for a substrate) was written in 1975 by Agarwal [2], where he expresses all the elements of the tensor in the form of a double integral over *k*_*x*_ and *k*_*y*_ as presented in Eq. (7) in Ref [1].

This double integral, as pointed out by Liu and McLeod, can be tedious to compute directly. Therefore, it is common practice to use cylindrical coordinates, as described by Rahmani et al. in 1997 [3]. The double integral is then transformed as follows:

(1) $$\begin{array}{ll} G_{i} & =\overset{+\infty }{\underset{-\infty }{\int }}\overset{+\infty }{\underset{-\infty }{\int }}f_{1}\left(k_{x},k_{y}\right)dk_{x}dk_{y}\\ & =\overset{\infty }{\underset{0}{\int }}\overset{2\pi }{\underset{0}{\int }}f_{2}\left(\theta ,k_{\rho }\right)k_{\rho }dk_{\rho }d\theta \\ & =\overset{\infty }{\underset{0}{\int }}f_{3}\left(k_{\rho }\right)k_{\rho }dk_{\rho }, \end{array}$$

with the following change of variables: *k*_*x*_ = *k*_*ρ*_ cos*θ* and *k*_*y*_ = *k*_*ρ*_ sin *θ* where *G*_*i*_ is one of the components of the Green’s tensor. The integration over the angle of rotation *θ* can be performed simply by introducing the Bessel functions [4] as explained in Refs. [3], [5], [6], [7].

As pointed out by Liu and McLeod, there remains the problem of the poles to resolve. In fact, these poles can be easily integrated by the following change of variables:

(2) $$k_{z}=\sqrt{\varepsilon k_{0}^{2}-k_{\rho }^{2}},$$

as proposed by Lukosz and Kunz in 1977 [8] or used more recently in the following Refs. [5], [9]. In this case we have *k*_*z*_d*k*_*z*_ = −*k*_*ρ*_d*k*_*ρ*_ and the components of the Green’s tensor read:

(3) $$G_{i}=\left(-\overset{\sqrt{\varepsilon }k_{0}}{\underset{0}{\int }}+\overset{i\infty }{\underset{0}{\int }}\right)f_{4}\left(k_{z}\right)dk_{z},$$

where the first integral is evaluated over the propagative waves, and the second one over the evanescent waves. With this change of variables, the function *f*_4_(*k*_*z*_) does not exhibit any pole anymore.

But there is an even more efficient way of integrating these poles and avoiding the oscillations inherent to the Sommerfield integrals. It is to use the residue theorem as presented by Paulus, Gay-Balmaz and Martin in 2000 [10]. This technique permits to avoid the poles due to the presence of guided waves in a multilayer. This seminal work explains in detail how to calculate very quickly the Green’s tensor of a multilayer. It begins by switching to cylindrical coordinates and by using the residue theorem to avoid all the poles and oscillations of the integrand, see Figure 4 of [10]. Then Paulus et al. explain how to calculate the integral for high *k* values to circumvent the weak decay and oscillations of the Bessel functions. The technique is very clever: the authors write the Bessel functions as a sum of two Hankel functions, which decay much faster than the Bessel functions without any oscillation, see Figure 5 of Ref. [10]. It’s the method described in this article that is used to calculate the multilayer Green function in the IFDDA code [11].

Note also that the authors use a Gauss–Kronrod method for integrating the Green’s tensor, in particular quadgk from Matlab. However, the quadgk function in Matlab does not use a nested sequence and the code refines adaptively the integration interval, which means recalculating new points and losing the points already calculated. Chaumet et al. in 2020 [12] suggest a Gauss–Kronrod–Patterson (GKP) method, which presents a nested quadrature rule [13] and permits to increase the integration convergence [14]. In this technique, one first estimates the integral with very few points then adds more points for reaching the targeted accuracy (without losing the previous ones). Note that the technique described by Paulus et al. with the GKP method requires usually less than 256 estimations of the integrand.

The authors suggest using the Green’s tensor in cylindrical coordinates, and interpolate it to a Cartesian grid. Chaumet et al. in 2020 [12] proposed the same approach and showed that it is necessary to interpolate the Green’s function with rational functions. Rational functions have the ability to model functions with poles [15] and permit an accurate approximation of the behavior of the Green tensor in the near field range.

There are also articles that calculate the Green’s tensor directly in Cartesian coordinates. In the present article, Liu and McLeod perform the integration with a trapezoidal mesh. This approach is very slow and not very efficient. Hence, Pincemin et al. in 1994 [16] performed the integration with a fast Fourier transform. This approach has two advantages: it is very fast to calculate and it yields the all the values of the Green function in one shot. However, the poles are always tricky to deal with, which is why we prefer the approach used by Paulus et al. Nevertheless, a comparison between the computation time taken when integrating in Cartesian or cylindrical coordinates can only be fair if both approaches are optimized to their maximum.

For example, with the elliptical cylinder above the substrate described by the author and the same discretization (*N* ≈ 200), it took 4 ms (on a Intel Xeon Silver 4214R CPU @ 2.4 GHz computer) to calculate the Green functions with the Paulus et al. technique as implemented in IF-DDA, while it took 31.4 s with Liu et al. technique (on a dual Intel Xeon CPU E5-2660 v3 @2.6 GHz processors computer). The efficiency of Paulus et al. technique is even more striking on large objects. With a sphere with a diameter of 12 μm represented by *N* = 2 × 10^6^ dipoles above a glass substrate the Green function calculation required only 171 s.

In conclusion, the method proposed by the authors for calculating efficiently the Green function of a multilayered system has been known for a long time and significantly improved in the meantime. In particular, the use of cylindrical coordinates is abundantly explained in the literature. The seminal article by Paulus et al., which explains how to calculate Green functions efficiently, addresses all the difficulties raised by the authors and proposes solutions that are faster than the ones proposed in Liu’s paper. Note that the work of Paulus et al. has been quoted more than 200 times.
